# Phonemic verbal fluency in non-WEIRD populations: Demographic differences in performance in the Controlled Oral Word Association Test-FAS

**DOI:** 10.4102/ajopa.v6i0.152

**Published:** 2024-05-24

**Authors:** Aline Ferreira-Correia, Hillary Banjo, Nicky Israel

**Affiliations:** 1 Department of Psychology, Faculty of Humanities, School of Human and Community Development, University of the Witwatersrand, Johannesburg, South Africa

**Keywords:** COWAT, verbal fluency, multilingualism, cognition, assessment

## Abstract

This study aimed to investigate whether age, level of education, gender, number of spoken languages, and the self-reported position of language within this multilingual experience predicted performance on the Controlled Oral Word Association Test (COWAT-FAS). Using a cross-sectional research design, the phonemic verbal fluency of a sample (*n* = 156) of healthy adults (ages 18–60 years) with different linguistic and educational backgrounds from a non-WEIRD (western, educated, industrialised, rich and democratic) context was assessed using the COWAT-FAS (including the F, A, S, total correct, repetition, incorrect, and total errors). Pearson’s correlations showed significant negative associations between age and most of the COWAT scores, including the total (*r* = –0.47; *p* < 0.01) and significant positive associations between years of education and all of the COWAT scores, including the total (*r* = 0.49; *p* < 0.01). The number of languages spoken was not significantly correlated with any of the COWAT scores, but multilinguals who identified English as a first language performed significantly better than those who identified English as a secondary language for several COWAT scores, including the total (*t*_154_ = 3.85; *p* < 0.001; *d* = 0.79). Age (*B* = –0.32; *p* < 0.001), years of education (*B* = 0.35; *p* < 0.001), and language position (*B* = –0.20; *p* < 0.01) also significantly predicted the COWAT total score (*r*^2^ = 0.38; *F* = 18.34; *p* < 0.001; f^2^ = 0.61). The implications of these findings for use of the COWAT-FAS in multilingual and non-WEIRD contexts are discussed.

**Contribution:**

This article supports the importance of understanding the role demographic variables play in cognitive performance and how they represent a source of bias in cognitive testing, particularly in the COWAT-FAS. It highlights how age, level of education, and the correspondence, or lack thereof, between first language and language of assessment, impacts phonemic fluency tasks. This knowledge may help to manage biases when conducting verbal fluency assessments with multilingual individuals and in non-WEIRD contexts.

## Introduction

Verbal fluency tasks assess an individual’s capacity to locate precise information under specific search criteria (Lezak et al., 2012), which are classified into two categories, namely semantic fluency and phonemic fluency. In order to measure phonemic fluency, test-takers are asked to produce as many words as they can that start with a particular letter, often F, A, or S; whereas semantic fluency is evaluated through the production of semantic group exemplars (typically names of animals) (Jebahi et al., 2020). Phonemic verbal fluency tests are one of the most widely used neuropsychological diagnostic methods because of the vulnerability of verbal fluency performance to certain cognitive impairments connected to lesions in the frontal lobe and deficits in the temporal lobe; they are therefore included in widely used batteries, screening tools, or as stand-alone tests (McDowd et al., 2011). Their capacity to discriminate between healthy ageing, mild cognitive impairment, and Alzheimer’s disease has been reported (McDonnell et al., 2020). However, the diagnostic power is reduced by the presence of biases that commonly arise when moderating factors are not taken into consideration, particularly while assessing populations outside of Western, educated, industrialised, rich and developed groups (i.e., non-WEIRD populations) (Ferreira-Correia & Cockroft, 2023).

Demographic factors such as age and education may have an impact on performance on phonemic fluency tests such as the Controlled Oral Word Association Test (COWAT-FAS) (Lubrini et al., 2022; Ross, 2003). It has been shown that age has a significant influence on verbal fluency tasks in some instances; however, results for these effects are inconsistent. On the one hand, standardised norms on the COWAT-FAS reveal an age dependent decline in performance, with younger adults performing better than older adults (Strauss et al., 2006). Tallberg et al. (2008) correlate this difference in performance between younger and older adults with a decline in information processing speed. They further assert that older adults have a lengthier reaction time before producing their first words and ultimately producing words at a slower rate. In contrast, other studies have been unable to identify an age-related difference in performance (Steiner et al., 2008).

The effects of gender on phonemic (letter) fluency have not been consistent. On the one hand, effects of gender on phonetic fluency have not been found (Mathuranath et al., 2003; Santos Nogueira et al., 2017). On the other hand, metanorms (Loonstra et al., 2001) and a metanalysis (Hirnstein et al., 2023) suggest a possible advantage of women over men in phonemic fluency. One review found that gender differences in this task may only be significant after 60 years of age (Rodrígue-Aranda & Martinussen, 2006).

Education has also been shown to have a significant influence on performance on phonemic verbal fluency tasks (Casals-Coll et al., 2013; Kosmidis et al., 2004). This advantage has been attributed to increased vocabulary exposure in highly educated people (Nogueira et al., 2016), which in turn has been linked to cognitive reserve, although with some variances related to race and ethnicity that may be linked to linguistic diversity (Avila et al., 2021; Rodríguez-Lorenzana et al., 2020). Specifically, educational disadvantage is of significance as it plays a major role in cognitive performance in countries and groups affected by major social inequalities (Manly et al., 2004), with South Africa being one example (Shuttleworth-Edwards, 2016).

The connection between verbal fluency and multilingualism is another area of research that has gained popularity over the years (Giovannoli et al., 2023). This is particularly important for South Africa as multilingualism is not just a linguistic phenomenon but also a central aspect of the cultural and educational experience of South Africans. The presence of two or more languages gives a perspective into how cognitive systems interact, which would not be seen if studies were limited to speakers of a single language, especially when that single language is highly proficient (Kroll & De Groot, 2009). One side of the debate argues that the constant need to manage the various known languages that are active in the brain and choosing the one appropriate for each unique occurrence enhances cognitive processes, especially executive functions (Giovannoli et al., 2020). The other side argues that multilingualism negatively impacts language production, as seen by slower reaction times and decreased lexical access accuracy (Shao et al., 2014) driven by increased executive control demands recruited by phonetic verbal fluency tasks (Patra et al., 2020). It is important to note that these debates are based on results derived from studies that primarily consist of samples that are from WEIRD societies, and it is often not considered whether these results are applicable across cultures (Cockcroft, 2022).

Hence, exploring possible biases in phonemic verbal fluency tests and particularly, the COWAT-FAS, constitutes a first step towards reducing measurement errors in the cognitive assessments of people from non-WEIRD populations, which are characterised by complex interactions between linguistic and educational experiences. With the aim of facilitating more accurate neuropsychological diagnosis, this study aimed to investigate whether age, level of education, gender, number of spoken languages, and the self-reported position of language within this multilingual experience predicted performance on the COWAT-FAS in a heterogeneous South African sample. The main hypothesis is that all these selected demographic variables would significantly predict the performance on this phonemic verbal fluency task.

## Methods

### Participants

The secondary data utilised in this study were derived from four primary studies with South African samples (Banjo, 2023; Ferreira-Correia et al., 2020; Lubbe, 2016; Motlhabane, 2016). All participants in these studies were healthy adults with no reported history of neurological, psychiatric, or metabolic illness. All participants were recruited using purposive (Cresswell & Plano Clark, 2011) and convenience sampling (Stratton, 2021). Participants were invited from the general community surrounding the researchers and through referrals from other participants (snowball sampling) (Sharma, 2017).

The final sample for this study consisted of 156 participants aged between 18 years and 60 years (Mean [*M*] = 36.95; standard deviation [s.d.] = 14.82). A majority of the sample identified as female (60.3%) and indicated that their first language was a language other than English (81.4%). Most participants spoke three or four languages (*M* = 4.19; s.d. = 1.43) and had more than 12 years of formal education (*M* = 13.11; s.d. = 2.32). There were 26 participants (16.7%) who had not completed high school (12 years of education). Additional demographic information is presented in Table 1.

**TABLE 1:** Demographic data for the sample (*n* = 156).

Variable	Category	*n*	%
Age (in years)	18–34	79	50.6
	35–60	77	49.4
Gender	Male	62	39.7
	Female	94	60.3
Formal education	7–12 years	69	44.2
	13–19 years	87	55.8
First language	English	29	18.6
	Other language	127	81.4
Number of languages spoken	1	1	0.6
	2	10	6.4
	3	41	26.3
	4	49	31.4
	5	33	21.2
	6	15	9.6
	7	3	1.9
	8–10	4	2.6

### Design

The study used a non-experimental and cross-sectional design (Field, 2017).

### Instruments

A demographic questionnaire (cf. Ferreira-Correia, 2019) was used to capture participants’ age, gender, years of education, occupation, and language experience in all studies. For the latter, the participant was asked to identify how many languages they knew and used and to rank them in terms of self-perceived proficiency. In addition to the COWAT scores, all the data were coded into a data set for this study.

The COWAT is a measure of phonemic verbal fluency in which the participants are asked to produce as many words as possible that begin with the specified letter (F, A, or S) within 1 min each (Lezak et al., 2012). During this task, the examinee is also asked not to use proper nouns, numbers, or words with different suffixes (Olabarrieta-Landa et al., 2017). The summation of different and correct words produced by the test-taker for all three-letter sets determines their overall score whereas inadmissible words such as repeated words, slang or proper names are counted as errors (Lezak et al., 2012).

The FAS version of the COWAT has an inter-rater reliability of 0.9, high test-retest reliability, and strong correlations between letter sets ranging from 0.85 to 0.94; it therefore has excellent psychometric properties within samples in the United States (Ross, 2003; Troyer, 2000). Thus far, there appear to have been no empirical efforts to produce letter sets adequate for the multicultural and multilingual South African context.

### Procedure

The data were obtained from four separate data collection processes that aimed to explore the relationship between multilingualism and cognitive function in South African samples (cf. Banjo, 2023; Ferreira-Correia, 2019; Lubbe, 2016; Motlhabane, 2016). The COWAT was part of different batteries (study-dependent) and administered in different locations, at the participant’s convenience. Appropriate assessment conditions were guaranteed, such as good ventilation and lighting, a table with a smooth surface, two chairs, and low noise levels.

All studies received ethics clearance. Appropriate testing conditions were adhered to as the questionnaires were administered in a well-lit room, with chairs, good ventilation, and low noise levels. The administration procedure of the demographic questionnaire and the COWAT was standardised and conducted face-to-face, except for 20 participants who opted for online administration because of coronavirus disease 2019 (COVID-19)-related precautions.

### Data analysis

Data for the study were captured in Microsoft Excel^©^ and analysed using IBM^®^ Statistical Package for the Social Sciences (SPSS^©^) version 28 (IBM Corp, 2021). Frequencies and percentages, means, standard deviations, and minimum and maximum scores were calculated to describe the nature of the data collected (Field, 2017). Skewness and kurtosis estimates and histograms indicated that the data for number of languages spoken, COWAT repetition errors, COWAT incomplete errors, and COWAT total errors were skewed, therefore a combination of parametric and non-parametric techniques were used to answer the research questions. Pearson’s and Spearman’s correlation coefficients were calculated to establish the nature of the relationships between age, years of education, number of languages spoken, and all COWAT scores; and independent *t*-tests and Mann–Whitney U tests were calculated to establish whether there were differences in COWAT scores between males and females and those who spoke English or another language as a first language (Field, 2017). Multiple regression models were run to establish if age, gender, years of education, number of languages spoken, and position of English predicted performance for the COWAT total score and the F, A, and S subscale scores (Field, 2017).

### Ethical considerations

This study used data from four separate projects that aimed to explore the relationship between multilingualism and cognitive performance (cf. Banjo, 2023; Ferreira-Correia, 2019; Lubbe, 2016; Motlhabane, 2016). Each project received ethical clearance from the University of the Witwatersrand Human Research Ethics Committees (Medical and Non-Medical) and followed the Helsinki Declaration and the Singapore Statement on Research Integrity (Resnik & Shamoo, 2011). Information about the study was provided in writing to all participants and discussed with them verbally (in person [*n* = 136] and online [*n* = 20]). Those who were willing to participate were required to give formal consent (M140872; MPSYC15/007/IH; MPSYC15/016/IH; MASPR/22/01).

## Results

As shown in Table 2, age was significantly and negatively associated with the COWAT total score (*r* = –0.472), and F (*r* = –0.363), A (*r* = –0.422), and S (*r* = –0.468) subscales as well as number of repetition errors (*r* = –0.164). Years of education was significantly and positively associated with the COWAT total score (*r* = 0.488), and F (*r* = 0.378), A (*r* = 0.430), and S (*r* = 0.497) subscales as well as repetition errors (*r* = 0.185), incorrect responses (*r* = 0.164), and total errors (*r* = 0.260). Number of languages spoken was not significantly related to any of the COWAT scores in the sample.

**TABLE 2:** Descriptive statistics and Pearson’s and Spearman’s correlation coefficients (*n* = 156).

Variable	AGE	YOE	NLS	CTT	CF	CA	CS	CRP†	CIN†	CTE†
Mean	36.95	13.11	4.19	31.07	11.26	8.21	11.60	0.48	0.48	0.96
Standard deviation	14.82	2.32	1.43	11.27	4.15	3.83	4.60	0.86	0.84	1.29
**Correlations**										
AGE	-	-	-	−0.472***	−0.363***	−0.422***	−0.468***	−0.164*	0.127	−0.026
YOE	-	-	-	0.488***	0.378***	0.430***	0.497***	0.185*	0.164*	0.260**
NLS^	-	-	-	−0.001	0.026	−0.046	0.004	0.067	−0.065	0.013

YOE, years of education; NLS, number of languages spoken; CTT, COWAT total score; CF, COWAT F subscale; CA, COWAT A subscale; CS, COWAT S subscale; CRP, COWAT repetition errors; CIN, COWAT incorrect responses; CTE, COWAT total errors.

* , *p* < 0.05;

** , *p* < 0.01;

*** , *p* < 0.001.

† , Spearman’s correlation coefficients.

There were significant differences in performance between the gender groups on the S subscale (*t*_154_ = –2.049; *p* = 0.042; *d* = 0.335) and for repetitions (*z* = –2.129; *p* = 0.033; *r* = 0.017). Participants who identified as female (*M*_*F*_ = 12.20) performed significantly better than those who identified as male (*M*_*M*_ = 10.68) on the S subscale, with a medium effect size. Female participants (*MR*_*F*_ = 83.67) also made significantly more repetition errors than male participants (*MR*_*M*_ = 70.66), although the effect size for this was small. There were no significant differences in performance between the gender groups for the COWAT total score, F and A subscales, incorrect responses, or total errors.

There were significant differences in performance between the position of English groups on the COWAT total score (*t*_154_ = 3.849; *p* < 0.001; *d* = 0.792), the F subscale (*t*_154_ = 2.540; *p* = 0.012; *d* = 0.523), the A subscale (*t*_154_ = 3.329; *p* = 0.001; *d* = 0.685), and the S subscale (*t*_154_ = 4.337; *p* < 0.001; *d* = 0.893), with medium effect sizes except for the S subscale, where the effect size was large. For the total score, those who spoke English as a first language (*M*_*E*_ = 38.03) significantly outperformed those who spoke another language as a first language (*M*_*A*_ = 29.48). Those who spoke English as a first language also performed significantly better than those who spoke another language as a first language for the F subscale (*M*_*E*_ = 13.00; *M*_*A*_ = 10.87); the A subscale (*M*_*E*_ = 10.28; *M*_*A*_ = 7.73); and the S subscale (*M*_*E*_ = 14.76; *M*_*A*_ = 10.87). There were no significant differences in performance between the position of English groups for repetition errors, incorrect responses, or total errors.

As shown in Table 3, for the COWAT total score, the regression model was significant with a large effect size, and the predictors accounted for 37.9% of the variation in the COWAT total score. Age, years of education, and position of English were significant predictors in the model. The regression model for the F subscale was significant with a moderate effect size. For this model, the predictors accounted for 22.3% of the variation in the F subscale scores and only age and years of education were significant predictors. For the A subscale, the regression model was significant with a large effect size, and the predictors accounted for 30.4% of the variation in the A subscale scores. Age, years of education, and position of English were significant predictors in the model. The regression model for the S subscale was significant with a large effect size. For this model, the predictors accounted for 40.2% of the variation in the S subscale scores and age, years, of education, and position of English were significant predictors.

**TABLE 3:** Regression models predicting the Controlled Oral Word Association Test total score and subscale scores (*n* = 156).

Criterion – Model	CTT		CF		CA		CS	
	b	t	b	t	b	t	b	t
Constant	31.071	43.002***	11.263	37.802***	8.205	31.536***	11.596	40.076***
AGE	−0.241	−4.521***	−0.072	−3.271***	−0.075	−3.879***	−0.092	−4.283***
GDR	0.079	0.053	−0.575	−0.925	0.066	0.121	0.563	0.932
YOE	1.697	4.974***	0.488	3.469***	0.524	4.267***	0.694	5.081***
NLS	−0.474	−0.208	0.251	0.268	−0.949	−1.158	0.207	0.227
PSE	−5.716	−2.888**	−1.435	−1.759	−1.470	−2.062*	−2.827	−3.566***
*R* ^2^	0.379	-	0.223	-	0.304	-	0.402	-
*F*	18.344***	-	8.622***	-	13.095***	-	20.127***	-
f^2^	0.610	-	0.287	-	0.437	-	0.672	-

CTT, COWAT total score; CF, COWAT F subscale; CA, COWAT A subscale; CS, COWAT S subscale; GDR, gender; YOE, years of education; NLS, number of languages spoken; PSE, position of English.

## Discussion

It is known that demographic variables, particularly age and education and, to a lesser extent, sex and gender, moderate performance on neuropsychological tests (Medina et al., 2021). Similarly, multilingualism (knowing and using more than one language) seems to significantly influence cognition (Quinteros Baumgart & Billick, 2018), although there is extensive debate around the nature and trajectory of this influence (Han et al., 2022). Understanding the interplay of these variables is particularly important for diverse and heterogenous populations, such as South Africa. In addition, exploring potential biases in neuropsychological tests such as the COWAT-FAS will allow practitioners and researchers to improve their judgement when working with these within these contexts. Therefore, this study aimed to investigate the relationships between COWAT-FAS scores and self-reported language use and proficiency, age, gender, and years of study in a sample of healthy participants. We hypothesised that all of the demographic variables would predict performance on a phonemic verbal fluency task (measured by the COWAT-FAS), which was the case with the exceptions of gender and number of languages spoken.

The study sample included a group of adult volunteers whose demographic characteristics reflected the heterogeneity of South Africa’s population, except for gender (which was biased towards females). Almost all our participants spoke more than one language and the majority identified an African language as their first language (Posel & Casale, 2011). Our sample was also heterogenous in terms of education – 17% of the sample had not completed high school and an additional 27% had not studied at the university level, which reflects the tragic educational inequalities that characterise South Africa (Taylor, 2019). Adults similar to our sample are more susceptible to biases in the interpretation of cognitive tests when inadequate norms are used (Ferreira-Correia & Cockcroft, 2023; Watts & Shuttleworth-Edwards, 2016).

The findings indicated that age was significantly and negatively related to and significantly predicted overall COWAT performance and performance on the COWAT subscales, with younger participants obtaining higher average scores than older participants. This is in support of the literature that reports an inverse relationship between age and performance in phonemic verbal fluency (Hatta et al., 2020; Rodríguez-Aranda & Martinussen, 2006; Strauss et al., 2006; Tallberg et al., 2008).

Also as anticipated, years of education was significantly and positively related to and positively predicted overall COWAT performance and performance on the COWAT subscales, with more highly educated participants obtaining higher average scores than less educated participants. The impact that education has on word production has been reported (Aki et al., 2022; İlkmen & Büyükişcan, 2022; Nogueira et al., 2016) and seems to be consistent across cultures and languages (Oberg & Ramírez, 2006).

Gender was not a significant predictor of performance for the overall COWAT score or the COWAT F and A subscales, but there were gender differences in performance on the S subscale. Participants who identified as female obtained higher average scores on the S subscale than participants who identified as male. Although this finding may be because of a selection bias in our sample, it also aligns well with the inconsistent pattern of findings that characterises gender differences in the literature. For example, a recent review revealed that females have better performance than males on phonemic fluency tasks (Hirnstein et al., 2023); however, other studies have suggested that males may outperform females on these types of tasks (Filippi et al., 2022) or that there are actually no significant differences in phonemic fluency between the genders (Lanting et al., 2009; Santos Nogueira, et al., 2016; Sokołowski et al., 2020; İlkmen & Büyükişcan, 2022). The findings in this study largely support the latter proposition of no differences based on gender, with the significant difference between the male and female participants for the S subscale possibly being an artefact of the sample, administration, or context.

It has been suggested that cognitive processing of multilinguals is different from that of bilinguals and monolinguals (Higby et al., 2013), specifically, empirical evidence indicates that speaking more than one language is detrimental to lexical access (Ivanova & Costa, 2008). Consequently, we hypothesised that the number of language participants reported speaking would have an impact on their phonetic verbal fluency, which was not the case. Total languages spoken was not significantly related to and did not significantly predict any of the COWAT scores. This supports the notion that additional languages may not additively increase difficulties with the COWAT-FAS. In contrast, whether the participants identified their first language as English or not was a significant predictor of performance for the overall COWAT score and the A and S subscales but not for the F subscale. There were also significant differences in performance between the position of English groups for the overall COWAT score and for all COWAT-FAS subscales, with participants who spoke English as a first language obtaining higher average scores than those who spoke another language as a first language. It can be hypothesised that participants who ranked English high in their multilingual experience might have a higher proficiency than those who ranked this language lower. This supports Luo et al. (2010) who found that monolinguals and low-vocabulary bilinguals have a lower performance in phonemic fluency tasks when compared with high-vocabulary bilinguals. These differences highlight a concern regarding potential language biases in the COWAT-FAS and support the need for the development of adapted letter sets and context-specific norms across different languages and linguistic groups.

Errors in the COWAT-FAS are an indication of potential impairment, and therefore have important diagnostic value (Strauss et al., 2006). Because of their clinical value, we also investigated how these were influenced by the selected demographic variables and self-assessed language experience in the study. We found that age was significantly and negatively related to the number of repetition errors made, with older participants making more repetition errors than younger participants. This was expected as older healthy adults display a decline in cognitive control and tend to make more errors in cognitive tasks (Larson et al., 2016).

In contrast, years of education were significantly and positively related to repetition errors, incorrect responses, and total errors, with more highly educated participants making more errors than less educated participants. This finding was unexpected because repetitions and incorrect words signal deficits with pre-frontal lobe functions such as self-monitoring and maintaining a cognitive set (Robinson et al., 2021). It is, however, possible that people with higher levels of education can achieve a better outcome on the COWAT-FAS by enhanced associative thinking, which could open the door to semantic associations that challenge the phonemic restriction imposed by the instruction, hence a lack of inhibition could facilitate the spread of activation but at the cost of incorrect responses (Alcock et al., 2023). Another possibility is the presence of unexplored interactions between quality of education and language of instruction, which can both affect test performance and vocabulary access in the language of assessment (Shuttleworth-Edwards & Truter 2023).

In the sample, participants who self-identified as female made significantly more repetition errors than participants who self-identified as male. Repetition errors, as a form of perseverative error, are seen as signalling a failure in inhibition and self-monitoring (Crawford et al., 1998). This finding does not align with those of Erden Aki et al. (2022), who reported no gender differences in repetition errors on a phonetic fluency task. Evidence regarding gender-based differences in cognition is, however, highly ambivalent and more work is needed to establish whether these have clinical significance.

There were no significant differences between the position of English groups for the different types of errors made and no relationships between the number of languages spoken and the errors made. This supports the relative independence of both perseverative and rule-break errors in the COWAT-FAS in terms of linguistic ability, thus enhancing the potential value of these as clinical indicators in multilingual populations.

This study provides valuable preliminary data regarding the use of the COWAT-FAS in non-WEIRD populations and addresses a critical gap in the existing research and assessment practices; however, there are some important limitations that should be acknowledged. We did not control for processing speed, which may have a mediating effect between age and verbal fluency performance (Elgamal et al., 2011). We did not explore the impact of quality of education and language of instruction on verbal fluency; future research in South Africa should consider exploring the interaction between these variables in terms of their influence on cognitive performance. Multilingualism is a natural phenomenon that is not susceptible to experimentation, hence no causal relationships can be established between the variables under investigation. There was no representation of monolinguals in our sample, which limits the exploration into the differences between monolingual and multilinguals. Our sample was also small and was not representative of the larger South African population despite a range of demographic characteristics present in the sample. The findings in this study thus cannot be generalised, although they do identify some important implications for further research.

### Implications and recommendations

There were biases in COWAT-FAS scores based on age, level of education, and position of English as a spoken language in our sample of healthy participants. This implies that caution is needed when administering and interpreting the COWAT in multicultural and multilingual contexts. Specifically, professionals using the COWAT-FAS should use norms stratified by age and years of education, and should be aware that this test may be biased against participants who identify as speaking English as a secondary language. The necessity of researchers striving to develop alternate letter sets and suitable norms for non-WEIRD populations is strongly supported, and these findings should be considered when exploring the criteria for stratification.

## Conclusion

The COWAT-FAS total score had a significant relationship with age, years of education, and position of English as a spoken language in our sample, suggesting possible biases that are important to consider when using the test in multilingual and multicultural populations. Further research is needed to establish the extent of these biases and to develop suitable letter sets and norms for appropriate clinical use of the COWAT-FAS in non-WEIRD populations.
